# Supplementary material for: Gut Microbiome Associated with the Psychoneurological Symptom Cluster in Patients with Head and Neck Cancers
Source: Cancers (Basel). 2020 Sep 6;12(9):2531. doi: 10.3390/cancers12092531 (PMC7563252; doi:10.3390/cancers12092531)
Supplement: Supplementary file 1 [file cancers-12-02531-s001.pdf]

Table S1. Functional Pathway Analysis of the Gut Microbiome based on PNS level.

Functional Pathway	PNS Group	n	Mean	SD	Non-corrected P-value	Corrected P-value
Lipid metabolism	high	13	3.4439	0.2443	0.0092	0.0469**
	low	13	3.1843	0.2568		
Glycan biosynthesis and metabolism	high	13	4.9069	0.3491	0.0003	0.0124**
	low	13	4.1443	0.5211		
Biosynthesis of other secondary metabolites	high	13	1.0622	0.1223	0.003	0.0216**
	low	13	0.9098	0.0854		
Xenobiotics biodegradation and metabolism	high	13	2.3641	0.0593	0.0204	0.0761*
	low	13	2.4539	0.1042		
Folding, sorting and degradation	high	13	2.9639	0.1194	0.003	0.0216**
	low	13	2.8087	0.1446		
Membrane transport	high	13	7.5459	1.1812	0.0037	0.0216**
	low	13	9.2448	1.4620		
Transport and catabolism	high	13	0.6840	0.0938	0.001	0.0213**
	low	13	0.5179	0.1203		
Cell motility	high	13	1.0211	0.3469	0.002	0.0216**
	low	13	1.4668	0.4824		
Digestive system	high	13	0.4197	0.0535	0.0037	0.0216**
	low	13	0.3236	0.0797		
Nervous system	high	13	0.1448	0.0135	0.0175	0.0719*
	low	13	0.1300	0.0156		
Cancers: Overview	high	13	0.0636	0.0057	0.0149	0.0683*
	low	13	0.0698	0.0065		

Note: This table lists the significant pathways associated with the PNS levels (high vs. low). n, sample size; PNS, psychoneurological symptoms; SD, standard deviation

*$p < 0.10$; ** $p < 0.05$
